# Effects of Statins on Renin–Angiotensin System

**DOI:** 10.3390/jcdd8070080

**Published:** 2021-07-09

**Authors:** Nasim Kiaie, Armita Mahdavi Gorabi, Željko Reiner, Tannaz Jamialahmadi, Massimiliano Ruscica, Amirhossein Sahebkar

**Affiliations:** 1 Research Center for Advanced Technologies in Cardiovascular Medicine, Tehran Heart Center, Tehran University of Medical Sciences, Tehran 1411713138, Iran; kiaien@mailfa.com (N.K.); gorabiam@mailfa.com (A.M.G.); 2 Department of Internal Diseases, School of Medicine, University Hospital Center Zagreb, Zagreb University, 10000 Zagreb, Croatia; zeljko.reiner@kbc-zagreb.hr; 3 Quchan Branch, Department of Food Science and Technology, Islamic Azad University, Quchan 9479176135, Iran; jamiat981@mums.ac.ir; 4 Department of Nutrition, Mashhad University of Medical Sciences, Mashhad 9177948564, Iran; 5 Department of Pharmacological and Biomolecular Sciences, Università degli Studi di Milano, 20133 Milan, Italy; massimiliano.ruscica@unimi.it; 6 Biotechnology Research Center, Pharmaceutical Technology Institute, Mashhad University of Medical Sciences, Mashhad 9177948564, Iran; 7 Applied Biomedical Research Center, Mashhad University of Medical Sciences, Mashhad 9177948564, Iran; 8 School of Medicine, The University of Western Australia, Perth 6009, Australia; 9 School of Pharmacy, Mashhad University of Medical Sciences, Mashhad 9177948564, Iran

**Keywords:** statins, renin–angiotensin system (RAS), hypertension

## Abstract

Statins, a class of drugs for lowering serum LDL-cholesterol, have attracted attention because of their wide range of pleiotropic effects. An important but often neglected effect of statins is their role in the renin–angiotensin system (RAS) pathway. This pathway plays an integral role in the progression of several diseases including hypertension, heart failure, and renal disease. In this paper, the role of statins in the blockade of different components of this pathway and the underlying mechanisms are reviewed and new therapeutic possibilities of statins are suggested.

## 1. Introduction

The renin–angiotensin system (RAS) and renin–angiotensin–aldosterone system (RAAS) are enzymatic pathways that contribute to the progression of cardiovascular disease (CVD) and CVD events and participate in CVD risk factors, e.g., hypertension. Besides CVD, this pathway contributes to several other biological actions, such as controlling blood flow in the ovary and uterus, fracture healing in the musculoskeletal system, and embryonic osteoblastosis [1].

This pathway starts with the secretion of renin (also named angiotensinogenase) by the juxtaglomerular cells in the kidney. Renin mediates the conversion of angiotensinogen into the inactive peptide angiotensin I, which then turns into an active hormone named angiotensin II (Ang II). This is precipitated by angiotensin-converting enzyme (ACE). The next step is the interaction of Ang II with its type 1 and type 2 receptors (AT1Rs and AT2Rs) [2,3]. Angiotensinogen can be transformed into Ang II directly by a serine protease named cathepsin G, which is released by neutrophils [4]. In some circumstances, ACE type 2 alters Ang II into Ang (1-7), which later interacts with AT2Rs and Mas Receptors (MasRs) [5]. Ang (1-7) can also be produced from Ang I by neprylisin (NEP) [6].

**Figure 1:**
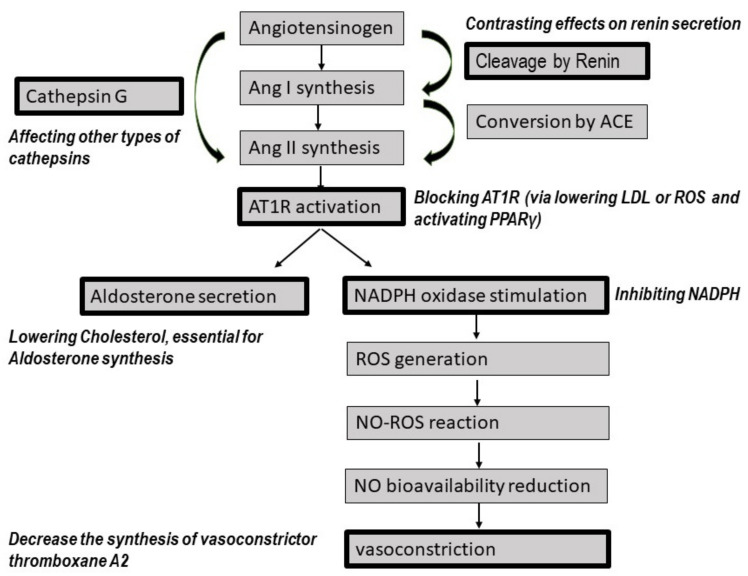
The effect of statins on RAS pathways. Statins affect renin secretion, aldosterone secretion, and vasoconstriction. Statins also block AT1Rs, increase ACE2 expression and NO release. The steps of pathways that are affected by statins are shown in black. Ang: angiotensin, ACE: angiotensin-converting enzyme, AT1Rs: angiotensin type 1 receptors, LDL: low-density lipoprotein, ROS: reactive oxygen species, PPAR: peroxisome proliferator-activated receptor, NADPH: nicotinamide adenine dinucleotide phosphate, NO: nitric oxide, NEP: neprylisin, MasR: Mas receptors.

AT1R activation by Ang II causes hypertension mainly as a result of the induction of aldosterone synthesis, vasoconstriction, and the activation of sympathetic nerves [7,8]. In contrast, AT2R activation stimulates vasodilation. Inhibition of cell growth, anti-inflammatory and anti-thrombotic responses are other results of AT2R activation by Ang (1-7) that prevent atherogenesis and atherosclerotic plaque progression [9,10]. MasR activation by Ang (1-7) also increases NO release and exerts an antiatherogenic response [11]. These interactions are presented in Figure 1.

Blocking the harmful RAS pathway (AT1R activation) has many clinical benefits. Common types of RAS blockers include angiotensin-converting enzyme inhibitors (ACEIs), angiotensin receptor blockers (ARBs), renin inhibitors, and aldosterone antagonists. These drugs can improve central nervous system disorders such as depression, while also having beneficial effects on CVD, including hypertension, atherosclerosis, myocardial infarction, chronic heart failure, and stroke, as well as in cases of renal disease [12,13,14]. Another potentially beneficial therapeutic effect of RAS suppression is its use in the treatment of patients with COVID-19 as a result of the recent disastrous pandemic [15,16].

Statins are the most well-known and widely used lipid-lowering drugs, and possess many important pleiotropic effects, including anti-apoptotic, antioxidant, anti-inflammatory, immunological, neuroprotective, and regenerative effects [17,18,19,20,21,22,23,24,25,26]. There are a number of different statins: lovastatin, simvastatin, atorvastatin, rosuvastatin, pitavastatin, fluvastatin, pravastatin, and mevastatin. They exert their effects mainly by inhibiting the activity of hydroxymethylglutaryl (HMG) CoA reductase [17,18]. Among these statins, the lipophilic ones exhibit higher polar interactions and binding to 3-hydroxy- 3-methylglutaryl-CoA reductase than those that are hydrophilic [27].

The results of studies indicating the beneficial synergistic effects of statins in combination with ACEIs and ARBs, as well as indicating the effects of statins on reducing blood pressure, suggest that statins have the potential to have significant effects on RAS pathway. Statins suppress this pathway by changing renin secretion, by influencing the synthesis of Ang I and Ang II, by suppressing AT1R activity, and by inhibiting aldosterone secretion [2,3,28]. Considering the importance of RAS regulation in the prevention and treatment of the serious diseases mentioned above, in addition to the widespread availability of statins, it is important to fully understand the mechanisms by which statins can either block or activate RAS pathways. Therefore, in this review, the effects of statins on different levels of RAS pathways are discussed.

## 2. Effect of Statins on RAS Pathways

### 2.1. The Effect of Statins on Renin Secretion

The RAS pathway is initiated by renin, a component cleaving angiotensinogen into Ang I, and is followed by synthesis of Ang II through conversion of Ang I by ACE. Renin secretion is controlled by specific stimulators and inhibitors. The most important stimulator of renin secretion is cAMP, which is activated by prostaglandins (I_2_ and E_2_), catecholamines (via β1-receptors), dopamine, norepinephrine (via β1-receptors), vasoactive intestinal peptide, calcitonin gene-related peptide (CGRP), and pituitary adenylate cyclase-activating peptide [29]. Neuropeptide Y is an inhibitor of cAMP [30]. 

Statins regulate renin secretion by affecting these cAMP activators. For example, in some studies, it has been shown that statins can induce prostaglandin I2 and E2 production by inhibiting geranylgeranylation, the process of attaching geranylgeranyl diphosphate to cysteine residues at the C-terminus of specific proteins, and inducing mRNA expression of COX-2, which metabolizes arachidonic acid into prostaglandins [31,32,33]. Induction of COX-2 expression by statins is driven by sterol-dependent and ERK1/2- and p38 MAPK-dependent pathways [34]. However, there are also conflicting results indicating that statins might suppress COX-2 and prostaglandin expression. Chen et al. reported that treatment of esophageal adenocarcinoma OE-19 cells and esophageal squamous cell carcinoma Eca-109 cells with different concentrations of simvastatin (15–75 μM) for 24 and 48 hours caused a downregulation of COX-2 and prostaglandin E2 expression [35]. Simvastatin (20 mg/kg/day for 30 days) also significantly decreased prostaglandin E_2_ production in rats [36] and atorvastatin (10 mg/kg) treatment of rats with periodontitis rats significantly downregulated COX-2 [37]. In another study, 96 h treatment with simvastatin reduced expression of COX-1 by suppressing fatty acid desaturase, but it increased COX-2 expression, which increased production of prostaglandins I2 and E2 [38].

Secretion of catecholamine, another activator of cAMP, is inhibited by statins as well. In several studies, simvastatin has been reported to decrease catecholamine synthesis in cultured bovine adrenal medullary cells, rat adrenal glands and perfused models of the isolated rat adrenal gland. The mechanism of this effect has been attributed to the elevation of nitric oxide (NO) levels, causing a blockade of nicotinic receptors, a necessary component of catecholamine secretion [39,40,41]. 

Proinflammatory effects of statins also indicate their role in decreasing the expression of CGRP which is another cAMP activator. For example, statins reduced bone morphogenetic protein (BMP)-induced CGRP expression in cultured sensory neurons in a concentration-dependent manner by a mechanism based on inhibiting smad1 phosphorylation and nuclear translocation [42].

Cyclic GMP (cGMP) is the second player in renin secretion, and is influenced by NO [43]. There is controversy surrounding the effect of NO/cGMP pathways. Some studies suggest that it might be an inhibitor, while others indicate that it might be a stimulator of renin secretion. Some studies have indicated that cGMP might have inhibitory effects on renin production by protein kinase G signaling [44].

However, a recent publication suggested that long-term administration of a cGMP stimulator (one dose daily for 7 days) in mice has no effect on renin regulation mediated by protein kinase G [45]. Other researchers have suggested that cGMP supports renin secretion due to inhibition of cAMP degradation by phosphodiesterases [46]. It has been shown that not only do NO/cGMP contribute directly to the increase in renin secretion, but that they also stimulate recruitment of renin-producing cells in the kidney [47]. These data, together with the effect of statins on NO level and eNOS expression, suggest a regulatory role of statins in renin secretion, although the mechanisms by which this is performed are not well known. Statins increase NO expression of endothelial cells through several mechanisms that have been previously reviewed [48]. Statins increase eNOS expression by activating PI3K/Akt, Rac1/AMPK and by inhibiting GGPP/Rho/ROCK pathways. They also increase NOS expression following the reduction of miR-221 or miR-222 expression. Statins can improve eNOS function due to CRP/GTPCH/ BH4 or Cav-1 pathways. Statins also block Nox expression via Rac1 or HO-1 pathways, causing increased NADPH expression and decreased reactive oxygen species (ROS) production, both of which result in reduced NO scavenging and improved NO bioavailability [48].

Atrial natriuretic peptide is another substance that inhibits renin secretion and is affected by statins. In human cultured endothelial cells, atorvastatin reduces natriuretic peptide, although this effect was not supported by the results of a study performed in vivo on patients with acute coronary syndrome [49]. However, a study showed that long-term (6 month) treatment of systolic heart failure patients with atorvastatin significantly reduces plasma natriuretic peptide levels [50]. The ability of atorvastatin to decrease serum natriuretic peptide levels was also observed in non-ST elevation myocardial infarction patients who were treated with high doses of atorvastatin (80 mg/day) and in acute myocardial infarction patients who were treated with atorvastatin within 48 h after the myocardial infarction [51,52]. Additionally, in a randomized placebo-controlled clinical trial, HIV-infected participants who were treated with rosuvastatin exhibited a reduction in plasma B-type natriuretic peptide [53]. These data, when put together, suggest that statins might increase renin secretion by decreasing the levels of its inhibitor.

Finally, statins affect cAMP activators in several ways. However, there are also data indicating that there is no specific effect of statins on these activators. For example, a clinical trial showed that treatment with simvastatin (40 mg/day) of hypertensive and hypercholesterolemic patients for 8 weeks did not have any effect on catecholamines, neuropeptide Y, aldosterone, and renin activity [8]. In addition to the effect of statins on cAMP, there are NO-related mechanisms confirming the results of the studies which have proven the effect of statins on cGMP. This reinforces the theory that renin secretion is affected by statins, but the exact mechanisms are still not clear.

### 2.2. The Effect of Statins on AT1R Expression and Activation

It has been shown that statins decrease AT1R mRNA expression in rat aortic vascular smooth muscle cells and endothelial cells, while statin withdrawal recovers AT1R expression [54,55]. In patients with hypercholesterolemia, statin therapy reduces AT1R density [55]. 

**Figure 2:**
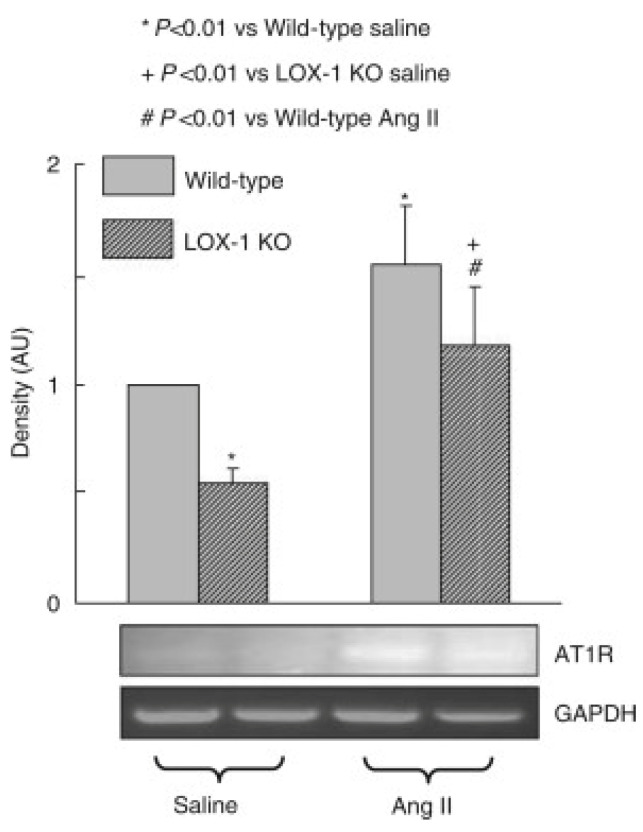
Expression of AT1R. Ang II-infused LOX-1 knockout mice showed less increase in the expression of AT1R (*n* = 5). AT1Rs: angiotensin type 1 receptors. Reproduced with permission from [58].

One suggested mechanism for AT1R downregulation might be the antioxidant effects of statins. For example, fluvastatin acts in a similar way to valsartan, an AT1R blocker, reducing superoxide production in ApoE−/− mice [56]. There is a linear relationship between the concentration of the oxidized form of low-density lipoprotein (LDL) in the plasma and the expression of AT1Rs in different cells such as vascular smooth muscle cells and platelets. The action of oxidized LDL on AT1R expression is mediated by its receptor, LOX-1, as presented in Figure 2, in which LOX-1 knock-out mice showed less expression of AT1Rs following Ang II infusion [57,58].

These data suggest that oxidation of LDL is an important step in the upregulation of AT1Rs. This is supported by the observation that administration of an oxidant to Sprague-Dawley rats increased AT1R expression [59,60]. The LDL-lowering effects of statins might be another mechanism explaining the reduced expression of AT1Rs and blockade of RAS pathways because, as stated previously, oxidized LDL is required for the upregulation of AT1Rs [61]. The third mechanism explaining the effect of statins on AT1Rs is based upon mediating the activation of peroxisome proliferator activated receptor gamma (PPARγ) in a similar way to some AT1R blockers [62,63]. There is a relationship between PPARγ and AT1Rs such that PPARγ inhibition upregulates AT1R expression and vice versa [64]. Sánchez-Aguilar et al. showed that PPARγ activation by rosiglitazone ligands decreased ACE expression, concentration of Ang II, and consequently AT1R activation [65]. It has been proven that one of the pleiotropic effects of statins is activation of PPARγ. For example, PPARγ expression was increased in primary human monocytes after treatment with atorvastatin [66]. In another study, treatment of HepG2 cells with seven different statins (atorvastatin, cerivastatin, fluvastatin, pitavastatin, pravastatin, rosuvastatin, and simvastatin) increased PPARγ mRNA expression in a dose-dependent manner [67]. In an in vivo study, simvastatin increased PPARγ expression in ApoE−/− mice with cardiac hypertrophy and fibrosis [68]. Two mechanisms for PPARγ activation by statins have been proposed. The first one is inhibition of the RhoA signaling, leading to ERK1/2 and p38 MAPK-dependent COX-2 expression, two cascades which increase a natural PPARγ ligand named 15d-PGJ2 [69]. The second one is binding of Leu331 and Tyr 334 residues of PPARα to statins so that the statin itself is considered to be a ligand for PPARα [70].

AT1Rs acquire an activated conformation following van der Waals interactions with Ang II [71,72], while other factors, such as mechanical stress, can activate these receptors [73,74]. The results of AT1R stimulation include activation of calcium signaling, ROS generation, vasoconstriction, and aldosterone secretion.

**Figure 3:**
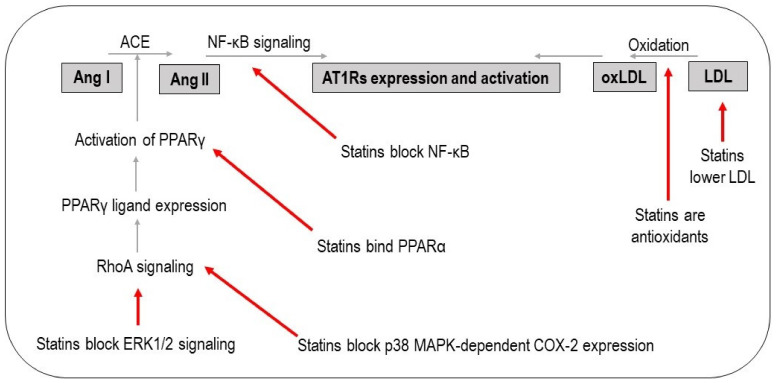
The effect of statins on AT1R expression and activation. LDL: low-density lipoprotein, oxLDL: oxidized low-density lipoprotein, AT1Rs: angiotensin type 1 receptors, ACE: angiotensin-converting enzyme, Ang: angiotensin, NF- κB: nuclear factor-κB, PPARγ: peroxisome proliferator activated receptor gamma.

Statins influence AT1R activation in addition to affecting its expression. Studies have shown that transcription factor nuclear factor-κB (NF-κB) signaling was necessary for Ang II-mediated activation of AT1Rs [75]. Statins decrease AT1R activity by blocking NF-κB [76,77]. The underlying mechanism is the lowering of cholesterol by statins, which first affects lymphotoxin β receptors and consequently the NF-κB signaling triggered by the lymphotoxin β receptors [78]. A study in hypertensive rats showed that pitavastatin decreased Ang II expression, and consequently, AT1R activation [79]. Some statins, including simvastatin and atorvastatin, decrease AT1R activation, resulting in the lower secretion of catecholamines [80]. As mentioned in the previous section, catecholamines are stimulating factors for the secretion of renin, an important substance in RAS pathway. Therefore, decreasing AT1R stimulation by statins suppresses RAS at the very beginning, i.e., it suppresses renin secretion. The pathways by which statins have effects on AT1R expression and activation are shown in Figure 3.

### 2.3. The Effect of Statins on AT2R and MasR Activation

It is known that Ang II in the RAS pathway acts on three types of receptors: AT1Rs, AT2Rs, and MasRs. While there have been several studies on the effect of statins on AT1R expression and activation, no study has shown a direct effect of statins on either AT2R or MasR expression and activation. One-month treatment of subjects at high risk for atherosclerosis with simvastatin reduced AT1R expression without affecting AT2Rs in monocytes [81]. In another study, rosuvastatin treatment of C57BL/6J mice with induced vascular injury did not alter the expression of AT2Rs [82]. It seems that statins can indirectly change the activation state of AT2Rs or MasRs by affecting ACE2, resulting in alterations in Ang (1-7) release [83]. For example, treatment of cholesterol-fed rabbits with atorvastatin increased the expression of ACE2 in the heart and kidney [84]. Treatment of rats with rosuvastatin after vascular balloon injury upregulated the expression of ACE2 and then the formation of Ang (1-7) [85]. In a clinical trial, it was shown that atorvastatin treatment of hypercholesterolemic subjects increased Ang (1-7)levels [86]. Additionally, Patel et al. found that downregulation or inhibition of AT1Rs could trigger activation of MasRs and AT2Rs. This suggests another possible indirect effect of statins on these two receptors [87].

### 2.4. The Effect of Statins on Aldosterone Secretion

The observation that aldosterone levels are associated with plasma concentration of lipoproteins such as low-density liporproteins (LDL) and high-density lipoproteins (HDL) suggests that statins, as drugs that have a primary effect on lipid metabolism, might also change the concentration of aldosterone [88]. Statins modulate aldosterone secretion and dysregulate RAS pathways. As Baudrand et al. showed, treatment with statins reduced aldosterone secretion in hypertensive and diabetic patients by about 33% and 26%, respectively, while levels of corticosterone, a precursor of aldosterone, remained unchanged after statin treatment, indicating that statins affect aldosterone production, rather than its metabolism [89]. A recent randomized, placebo-controlled, double-blinded study on 100 healthy individuals also showed that treatment with simvastatin resulted in reduced aldosterone levels [90].

The inhibitory effect of statins on aldosterone secretion depends on the lipophilicity of statins, because of the different affinities of statins for adrenal tissue, as well as upon the dose of statins [89]. The effect of statins on aldosterone inhibition is attributed to the reduced availability of cholesterol, as an essential element for steroid hormone production, as well as for suppressing the expression on AT1Rs in adrenal glands, which causes a reduction of Ang II interactions with AT1Rs, and finally the reduction of aldosterone synthesis [91].

### 2.5. The Effect of Statins on Vasoconstriction

It is well known that vasoconstriction is a consequence of RAS pathway activation. As presented in Figure 1, following AT1R activation, NADPH oxidase is stimulated, and ROS are produced. They later react with NO and reduce NO bioavailability. Finally, vasoconstriction occurs as a result of the reduced availability of NO. Since statins break down the RAS pathway, this can reduce vasoconstriction. Additionally, statins act directly on vasoconstriction by affecting vasoconstrictors like thromboxane A2. According to a study by Pignatelli et al., administration of 40 mg of atorvastatin in hypercholesterolemic patients inhibited the formation of platelet thromboxane A2 [92]. In an in vitro study, atorvastatin (20 µmol) reduced thromboxane A2 synthesis in platelets [93]. Similar results were obtained in a clinical study in which treatment with simvastatin (40 mg/day) in aspirin-resistant patients with a higher risk of cardiovascular disease reduced thromboxane A_2_ [94].

### 2.6. The Effect of Statins on Sympathetic Activation

The sympathetic nervous system, which is responsible for the body’s responses to stress, is activated following AT1R activation. Some studies have confirmed the effect of statins on the sympathetic system. In one study, treatment with simvastatin for one month in heart failure patients decreased the sympathetic activity of resting muscle [7]. Such a reduction in sympathetic activity has also been reported in patients with hypertension, chronic kidney disease and heart failure after treatment with simvastatin or atorvastatin [8,95,96].

The effect of statins on the sympathetic system is mediated by lowering ROS generation and then downregulating AT1Rs, as presented in Figure 1. Other mechanisms have also been suggested, including the induction of structural changes in the carotid arteries, such as increased numbers of elastic lamellae, decreased intima thickness and increased baroreceptor sensitivity, by simvastatin, resulting in a reduction of sympathetic system activity [97].

Statins might also modulate adrenoreceptors that regulate stimulation of sympatheric nervous system [98]. There is evidence suggesting that statins affect adrenoreceptors directly through unprenylation of Gγ subunits in adrenoreceptors or indirectly through inhibiting ERK activation [99,100,101]. Cerivastatin and simvastatin inhibited the stimulation of β-adrenergic receptors in adult rat ventricular myocytes. Atorvastatin also increased β-adrenergic receptor density in rat cardiac myocytes and α(1D)-adrenoceptor mRNA expression in the rat aorta [102,103]. In diabetic rats receiving oral atorvastatin, the protein expression of β1-adrenoceptor increased and β1/β3-adrenoceptor ratio changed [104]. Atorvastatin and simvastatin also improved cognitive performance in a mouse model of spatial memory by modulating β-adrenergic receptors [105].

A comparison between a lipophilic statin (atorvastatin) and a hydrophilic statin (rosuvastatin) showed that the effect of hydrophilic statins on sympathetic nerves was more significant due to the easier transfer of lipophilic statin through the blood–brain barrier [106].

## 3. RAS-Mediated Effects of Statins on COVID-19 Treatment

Since statins also have antiviral effects, they could also be beneficial in COVID-19 management [107]. It is well known that they have pleiotropic effects such as maintaining normal endothelial function, and attenuating inflammatory mediators. These anti-inflammatory and anti-thrombotic effects not only prevent myocardial injury, they may also have an effect in reducing mortality and endo tracheal intubation rates. Viruses enter the plasma membrane through receptors that are found on lipid rafts rich in cholesterol and sphingolipids. In a recent computational docking analysis, statins formed a strong bond with SARS-CoV-2 protease (Mpro) compared to other protease inhibitors, indicating a plausible mechanism by which statins contribute to SARS-CoV-2 replication [108,109,110]. 

In addition to the above mechanisms, the effect of statins in the treatment of patients with COVID-19 by regulating RAS pathways has attracted attention. Namely, there is recent evidence suggesting that RAS pathways are also involved in the progression of COVID-19 [111]. This evidence was confirmed when RAS inhibition in COVID-19 patients resulted in reduced IL-6 levels in peripheral blood, increased number of CD3 and CD8 T cells, and decreased peak viral load [112]. One component of RAS pathways that acts as an entry receptor for SARS-CoV-2 is ACE2 [113]. SARS-CoV-2 by binding to ACE2 receptors enters into the cell through the fusion of its membrane with that of the cell. Hence, it downregulates these receptors. The loss of ACE2 receptor activity from the external site of the membrane leads to less angiotensin II inactivation and less generation of Ang (1-7) [114]. In the lungs, such dysregulation could favor the progression of inflammatory and thrombotic processes triggered by local angiotensin II hyperactivity unopposed by Ang (1-7) [115]. The decrease in ACE2 activity by SARS-CoV-2 can unleash a cascade of injurious effects through a heightened imbalance in the actions of the products of ACE vs ACE2 [116].

ACE2 overexpression caused by statins could be beneficial for COVID-19 improvement because it has beneficial effect on ACE2/MasR axis of the RAS pathway, and thereby mediates severe inflammation in patients [117,118,119,120]. 

Therefore, the antiviral properties of statins, in addition to their inhibitory effects on RAS pathways by blocking AT1R, as well as their supportive role in ACE2/MasRs activation, suggest that they might be an effective additional therapy for COVID-19 patients. 

## 4. Potential Roles of Statins on Other RAS Elements

In-depth analysis of the mechanisms and molecules involved in the expression of cathepsin G and the role of statins in those mechanisms could be important for finding other potential mechanisms for the effect of statins on RAS suppression. The direct synthesis of Ang II from angiotensinogen occurs due to the activity of cathepsin G. There is no evidence so far that statins have an effect on this enzyme, but it is known that statins affect both polymorphonuclear neutrophils, cells which express cathepsin G gene, and other types of cathepsins. Cathepsins affect each other’s expression profiles [121]. Therefore, if statins have an effect on other types of cathepsins, it is highly probable that cathepsin G would also be regulated. There are data suggesting that statins do have an effect on cathepsins. Simvastatin decreases the activity of cathepsin B and cathepsin A and pravastatin increases the activity of cathepsin B. The activities of cathepsin H and cathepsin L are also reduced by treatment with statins [122,123,124]. It seems that these effects are dependent upon the hydrophilicity of statins, which determines the tissue distribution of statins, their bioavailability, and subsequent uptake by cells [123]. Statins affect cathepsins by down-regulating legumain mRNA, another protease involved in maturation of cathepsins, as well as regulation of glucose, which also affects the activity of cathepsins [125]. Additionally, the immunomodulatory properties of statins suggest that statins can affect cathepsin G production indirectly by reducing the number of neutrophils. Studies have shown that simvastatin administration in a rat model of intracerebral hemorrhage reduces the neutrophil count by increasing the expression of apoptotic related proteins [126]. The apaptotic effect of simvastatin (40 mg/day) treatment on neutrophils has also been reported in patients undergoing coronary surgery with cardiopulmonary bypass [127].

Another substance suggesting that statins might have an effect on RAS is NEP. Yamamoto et al. showed that simvastatin and atorvastatin increase secretion of soluble NEP in rat brain following induction of morphological changes in astrocytes and activation of MAPK/Erk1/2 pathways [128]. Since NEP contributes to the transformation of Ang I into Ang (1-7) in RAS, further studies on the role of statins on NEP release will increase our knowledge of the regulatory roles of statins in RAS pathways. 

## 5. Conclusions

In this review, we showed that statins affect several components of RAS pathways, and that the effect of statins is mainly the inhibition of the harmful axis (Ang II/AT1R) and the activation of the beneficial axis (Ang (1-7)/MasRs). Statins alter renin secretion, and therefore, they have effects on the synthesis of Ang I and Ang II. Statins can also suppress AT1R activity while improving AT2Rs and MasRs activity. The inhibitory effect of statins on NADPH and aldosterone secretion, as well as its supporting role in AT2Rs and MasRs activation is important in prevention of hypertension. In general, statins could be effective in the treatment of changes caused by the activation of RAS pathways.

## Data Availability

Not applicable.

## References

[1] Tsukamoto I., Inoue S., Teramura T., Takehara T., Ohtani K., Akagi M. (2013). Activating types 1 and 2 angiotensin II receptors modulate the hypertrophic differentiation of chondrocytes. FEBS Open Bio.

[2] Nickenig G. (2004). Should angiotensin II receptor blockers and statins be combined?. Circulation.

[3] Spindler S.R., Mote P.L., Flegal J.M. (2016). Combined statin and angiotensin-converting enzyme (ACE) inhibitor treatment increases the lifespan of long-lived F1 male mice. Age.

[4] Rykl J., Thiemann J., Kurzawski S., Pohl T., Gobom J., Zidek W., Schlüter H. (2006). Renal cathepsin G and angiotensin II generation. J. Hypertens..

[5] Sampaio W.O., Souza dos Santos R.A., Faria-Silva R., da Mata Machado L.T., Schiffrin E.L., Touyz R.M. (2007). Angiotensin-(1-7) through receptor Mas mediates endothelial nitric oxide synthase activation via Akt-dependent pathways. Hypertension.

[6] Santos R.A. (2014). Angiotensin-(1–7). Hypertension.

[7] Deo S.H., Fisher J.P., Vianna L.C., Kim A., Chockalingam A., Zimmerman M.C., Zucker I.H., Fadel P.J. (2012). Statin therapy lowers muscle sympathetic nerve activity and oxidative stress in patients with heart failure. Am. J. Physiol. Heart Circ. Physiol..

[8] Lewandowski J., Siński M., Bidiuk J., Abramczyk P., Dobosiewicz A., Ciarka A., Gaciong Z. (2010). Simvastatin reduces sympathetic activity in men with hypertension and hypercholesterolemia. Hypertens. Res..

[9] Montezano A.C., Cat A.N.D., Rios F.J., Touyz R.M. (2014). Angiotensin II and vascular injury. Curr. Hypertens. Rep..

[10] Silva G.M., França-Falcão M.S., Calzerra N.T.M., Luz M.S., Gadelha D.D.A., Balarini C.M., Queiroz T.M. (2020). Role of Renin-Angiotensin System Components in Atherosclerosis: Focus on Ang-II, ACE2, and Ang-1–7. Front. Physiol..

[11] Pignone A., Del Rosso A., Brosnihan K.B., Perfetto F., Livi R., Fiori G., Guiducci S., Cinelli M., Rogai V., Tempestini A. (2007). Reduced circulating levels of angiotensin-(1–7) in systemic sclerosis: A new pathway in the dysregulation of endothelial-dependent vascular tone control. Ann. Rheum. Dis..

[12] Vian J., Pereira C., Chavarria V., Köhler C., Stubbs B., Quevedo J., Kim S.-W., Carvalho A.F., Berk M., Fernandes B.S. (2017). The renin–angiotensin system: A possible new target for depression. BMC Med..

[13] Oliveros E., Oni E.T., Shahzad A., Kluger A.Y., Lo K.B., Rangaswami J., McCullough P.A. (2020). Benefits and Risks of Continuing Angiotensin-Converting Enzyme Inhibitors, Angiotensin II Receptor Antagonists, and Mineralocorticoid Receptor Antagonists during Hospitalizations for Acute Heart Failure. Cardiorenal Med..

[14] Ma T.K.W., Kam K.K.H., Yan B.P., Lam Y.-Y. (2010). Renin-angiotensin-aldosterone system blockade for cardiovascular diseases: Current status. Br. J. Pharmacol..

[15] Momtazi-Borojeni A.A., Banach M., Reiner Ž., Pirro M., Bianconi V., Al-Rasadi K., Sahebkar A. (2021). Interaction Between Coronavirus S-Protein and Human ACE2: Hints for Exploring Efficient Therapeutic Targets to Treat COVID-19. Angiology.

[16] Melissa Hallow K., Dave I. (2021). RAAS Blockade and COVID-19: Mechanistic Modeling of Mas and AT1 Receptor Occupancy as Indicators of Pro-Inflammatory and Anti-Inflammatory Balance. Clin. Pharmacol. Ther..

[17] Cai J., Yu X., Zhang B., Zhang H., Fang Y., Liu S., Liu T., Ding X. (2014). Atorvastatin improves survival of implanted stem cells in a rat model of renal ischemia-reperfusion injury. Am. J. Nephrol..

[18] Aktas O., Albrecht P., Hartung H.P. (2016). Optic neuritis as a phase 2 paradigm for neuroprotection therapies of multiple sclerosis: Update on current trials and perspectives. Curr. Opin. Neurol..

[19] Reiner Ž. (2013). Statins in the primary prevention of cardiovascular disease. Nat. Rev. Cardiol..

[20] Afshari A.R., Mollazadeh H., Henney N.C., Jamialahmad T., Sahebkar A. (2021). Effects of statins on brain tumors: A review. Semin. Cancer Biol..

[21] Bahrami A., Bo S., Jamialahmadi T., Sahebkar A. (2020). Effects of 3-hydroxy-3-methylglutaryl coenzyme A reductase inhibitors on ageing: Molecular mechanisms. Ageing Res. Rev..

[22] Gorabi A.M., Kiaie N., Pirro M., Bianconi V., Jamialahmadi T., Sahebkar A. (2020). Effects of statins on the biological features of mesenchymal stem cells and therapeutic implications. Heart Fail. Rev..

[23] Mollazadeh H., Tavana E., Fanni G., Bo S., Banach M., Pirro M., von Haehling S., Jamialahmadi T., Sahebkar A. (2021). Effects of statins on mitochondrial pathways. J. Cachexia Sarcopenia Muscle.

[24] Reiner Ž., Hatamipour M., Banach M., Pirro M., Al-Rasadi K., Jamialahmadi T., Radenkovic D., Montecucco F., Sahebkar A. (2020). Statins and the Covid-19 main protease: In silico evidence on direct interaction. Arch. Med. Sci..

[25] Sahebkar A., Serban C., Ursoniu S., Mikhailidis D.P., Undas A., Lip G.Y.H., Bittner V., Ray K.K., Watts G.F., Kees Hovingh G. (2016). The impact of statin therapy on plasma levels of von Willebrand factor antigen: Systematic review and meta-analysis of Randomised placebo-controlled trials. Thromb. Haemost..

[26] Serban C., Sahebkar A., Ursoniu S., Mikhailidis D.P., Rizzo M., Lip G.Y.H., Kees Hovingh G., Kastelein J.J.P., Kalinowski L., Rysz J. (2015). A systematic review and meta-analysis of the effect of statins on plasma asymmetric dimethylarginine concentrations. Sci. Rep..

[27] Istvan E.S., Deisenhofer J. (2001). Structural mechanism for statin inhibition of HMG-CoA reductase. Science.

[28] Ruszkowski P., Masajtis-Zagajewska A., Nowicki M. (2019). Effects of combined statin and ACE inhibitor therapy on endothelial function and blood pressure in essential hypertension—A randomised double-blind, placebo controlled crossover study. J. Renin Angiotensin Aldosterone Syst. JRAAS.

[29] Kurtz A. (2012). Control of Renin Synthesis and Secretion. Am. J. Hypertens..

[30] Herring N., Tapoulal N., Kalla M., Ye X., Borysova L., Lee R., Dall’Armellina E., Stanley C., Ascione R., Lu C.-J. (2019). Neuropeptide-Y causes coronary microvascular constriction and is associated with reduced ejection fraction following ST-elevation myocardial infarction. Eur. Heart J..

[31] Levine L. (2003). Statins stimulate arachidonic acid release and prostaglandin I. Lipids Health Dis..

[32] Hazra S., Lee G., Grant J., Walser T., Prasad S., Larsen J.E., Minna J., Dubinett S.M. (2010). Abstract B57: Modulation of prostaglandin E2 by statins in human bronchial epithelial cells harboring K-ras mutation: The potential advantage of combination therapy. Cancer Prev. Res..

[33] Mouawad C.A., Mrad M.F., El-Achkar G.A., Abdul-Sater A., Nemer G.M., Creminon C., Lotersztajn S., Habib A. (2016). Statins Modulate Cyclooxygenase-2 and Microsomal Prostaglandin E Synthase-1 in Human Hepatic Myofibroblasts. J. Cell. Biochem..

[34] Yano M., Matsumura T., Senokuchi T., Ishii N., Murata Y., Taketa K., Motoshima H., Taguchi T., Sonoda K., Kukidome D. (2007). Statins Activate Peroxisome Proliferator-Activated Receptor γ Through Extracellular Signal-Regulated Kinase 1/2 and p38 Mitogen-Activated Protein Kinase–Dependent Cyclooxygenase-2 Expression in Macrophages. Circ. Res..

[35] Chen Y., Li L.-B., Zhang J., Tang D.-P., Wei J.-J., Zhuang Z.-H. (2018). Simvastatin, but not pravastatin, inhibits the proliferation of esophageal adenocarcinoma and squamous cell carcinoma cells: A cell-molecular study. Lipids Health Dis..

[36] Nassar P.O., Nassar C.A., Guimarães M.R., Aquino S.G., Andia D.C., Muscara M.N., Spolidorio D.M.P., Rossa C., Spolidorio L.C. (2009). Simvastatin therapy in cyclosporine A-induced alveolar bone loss in rats. J. Periodontal Res..

[37] Araújo R.F.d.J., Souza T.O., Moura L.M.d., Torres K.P., Souza L.B.d., Alves M.d.S.C.F., Rocha H.O., de Araújo A.A. (2013). Atorvastatin Decreases Bone Loss, Inflammation and Oxidative Stress in Experimental Periodontitis. PLoS ONE.

[38] Grunwald S.A., Popp O., Haafke S., Jedraszczak N., Grieben U., Saar K., Patone G., Kress W., Steinhagen-Thiessen E., Dittmar G. (2020). Statin-induced myopathic changes in primary human muscle cells and reversal by a prostaglandin F2 alpha analogue. Sci. Rep..

[39] Koh Y.Y., Lim D.Y. (2016). Os 31-08 inhibitory effects of simvastatin on catecholamine secretion from the adrenal medulla. J. Hypertens..

[40] Matsuda T., Toyohira Y., Ueno S., Tsutsui M., Yanagihara N. (2008). Simvastatin Inhibits Catecholamine Secretion and Synthesis Induced by Acetylcholine via Blocking Na^+^ and Ca^2+^ Influx in Bovine Adrenal Medullary Cells. J. Pharmacol. Exp. Ther..

[41] Koh Y.K., Kim K.H., Choi M.S., Koh Y.Y., Lim D.Y. (2018). Simvastatin reduces adrenal catecholamine secretion evoked by stimulation of cholinergic nicotinic and angiotensinergic AT(1) receptors. Arch. Pharm. Res..

[42] Bucelli R.C., Gonsiorek E.A., Kim W.-Y., Bruun D., Rabin R.A., Higgins D., Lein P.J. (2008). Statins Decrease Expression of the Proinflammatory Neuropeptides Calcitonin Gene-Related Peptide and Substance P in Sensory Neurons. J. Pharmacol. Exp. Ther..

[43] Garmaroudi F.S., Handy D.E., Liu Y.-Y., Loscalzo J. (2016). Systems Pharmacology and Rational Polypharmacy: Nitric Oxide−Cyclic GMP Signaling Pathway as an Illustrative Example and Derivation of the General Case. PLoS Comput. Biol..

[44] Wagner C., Pfeifer A., Ruth P., Hofmann F., Kurtz A. (1998). Role of cGMP-kinase II in the control of renin secretion and renin expression. J. Clin. Investig..

[45] Schramm A., Schweda F., Sequeira-Lopez M.L.S., Hofmann F., Sandner P., Schlossmann J. (2019). Protein Kinase G Is Involved in Acute but Not in Long-Term Regulation of Renin Secretion. Front. Pharmacol..

[46] Beierwaltes W.H. (2006). cGMP stimulates renin secretion in vivo by inhibiting phosphodiesterase-3. Am. J. Physiol. Renal Physiol..

[47] Neubauer B., Machura K., Kettl R., Lopez Maria Luisa S.S., Friebe A., Kurtz A. (2013). Endothelium-Derived Nitric Oxide Supports Renin Cell Recruitment Through the Nitric Oxide–Sensitive Guanylate Cyclase Pathway. Hypertension.

[48] Gorabi A.M.K.N., Hajighasemi S., Banach M., Penson P.E., Jamialahmadi T., Sahebkar A. (2019). Statin-Induced Nitric Oxide Signaling: Mechanisms and Therapeutic Implications. J. Clin. Med..

[49] Prickett T., Troughton R., Espiner E. (2020). Effect of statin therapy on plasma C-type Natriuretic Peptides and Endothelin-1 in males with and without symptomatic coronary artery disease. Sci. Rep..

[50] Abulhul E., McDonald K., Martos R., Phelan D., Spiers J.P., Hennessy M., Baugh J., Watson C., O'Loughlin C., Ledwidge M. (2012). Long-term statin therapy in patients with systolic heart failure and normal cholesterol: Effects on elevated serum markers of collagen turnover, inflammation, and B-type natriuretic peptide. Clin. Ther..

[51] Shehata M., Samir A., Dardiri M. (2017). Prognostic impact of intensive statin therapy on N-terminal pro-BNP level in non-ST-segment elevation acute myocardial infarction patients. J. Interv. Cardiol..

[52] Teshima Y., Yufu K., Akioka H., Iwao T., Anan F., Nakagawa M., Yonemochi H., Takahashi N., Hara M., Saikawa T. (2009). Early atorvastatin therapy improves cardiac function in patients with acute myocardial infarction. J. Cardiol..

[53] Dirajlal-Fargo S., Kinley B., Jiang Y., Longenecker C.T., Hileman C.O., Debanne S., McComsey G.A. (2015). Statin therapy decreases N-terminal pro-B-type natriuretic peptide in HIV: Randomized placebo-controlled trial. AIDS.

[54] Castejon A.M., Zollner E., Tristano A.G., Cubeddu L.X. (2007). Upregulation of angiotensin II-AT1 receptors during statin withdrawal in vascular smooth muscle cells. J. Cardiovasc. Pharmacol..

[55] Nickenig G., Bäumer A.T., Temur Y., Kebben D., Jockenhövel F., Böhm M. (1999). Statin-Sensitive Dysregulated AT1 Receptor Function and Density in Hypercholesterolemic Men. Circulation.

[56] Li Z., Iwai M., Wu L., Liu H.W., Chen R., Jinno T., Suzuki J., Tsuda M., Gao X.Y., Okumura M. (2004). Fluvastatin enhances the inhibitory effects of a selective AT1 receptor blocker, valsartan, on atherosclerosis. Hypertension.

[57] Li D., Saldeen T., Romeo F., Mehta Jawahar L. (2000). Oxidized LDL Upregulates Angiotensin II Type 1 Receptor Expression in Cultured Human Coronary Artery Endothelial Cells. Circulation.

[58] Nazzaro P., Manzari M., Merlo M., Triggiani R., Scarano A., Ciancio L., Pirrelli A. (1999). Distinct and Combined Vascular Effects of ACE Blockade and HMG-CoA Reductase Inhibition in Hypertensive Subjects. Hypertension.

[59] Banday A.A., Lokhandwala M.F. (2008). Oxidative stress-induced renal angiotensin AT1 receptor upregulation causes increased stimulation of sodium transporters and hypertension. Am. J. Physiol. Renal Physiol..

[60] Hu C., Kang B.Y., Megyesi J., Kaushal G.P., Safirstein R.L., Mehta J.L. (2009). Deletion of LOX-1 attenuates renal injury following angiotensin II infusion. Kidney Int..

[61] Strehlow K., Wassmann S., Böhm M., Nickenig G. (2000). Angiotensin AT1 receptor over-expression in hypercholesterolaemia. Ann. Med..

[62] Benson S.C., Pershadsingh H.A., Ho C.I., Chittiboyina A., Desai P., Pravenec M., Qi N., Wang J., Avery M.A., Kurtz T.W. (2004). Identification of telmisartan as a unique angiotensin ii receptor antagonist with selective pparγ–modulating activity. Hypertension.

[63] Schupp M., Janke J., Clasen R., Unger T., Kintscher U. (2004). Angiotensin Type 1 Receptor Blockers Induce Peroxisome Proliferator-Activated Receptor- Activity. Circulation.

[64] Tiyerili V., Becher U.M., Camara B., Yildirimtürk C., Aksoy A., Kebschull M., Werner N., Nickenig G., Müller C. (2015). Impact of peroxisome proliferator-activated receptor γ on angiotensin II type 1 receptor-mediated insulin sensitivity, vascular inflammation and atherogenesis in hypercholesterolemic mice. Arch. Med. Sci. AMS.

[65] Sánchez-Aguilar M., Ibarra-Lara L., Mondragón L., Rubio-Ruiz M., Aguilar-Navarro A., Zamorano A., Ramírez-Ortega M., Hernández G., Sánchez-Mendoza A. (2019). Rosiglitazone, a Ligand to PPAR γ, Improves Blood Pressure and Vascular Function through Renin-Angiotensin System Regulation. PPAR Res..

[66] Grip O., Janciauskiene S., Lindgren S. (2002). Atorvastatin activates PPAR-γ and attenuates the inflammatory response in human monocytes. Inflamm. Res..

[67] Seo M., Inoue I., Ikeda M., Nakano T., Takahashi S., Katayama S., Komoda T. (2008). Statins Activate Human PPARalpha Promoter and Increase PPARalpha mRNA Expression and Activation in HepG2 Cells. PPAR Res..

[68] Qin Y.-W., Ye P., He J.-Q., Sheng L., Wang L.-Y., Du J. (2010). Simvastatin inhibited cardiac hypertrophy and fibrosis in apolipoprotein E-deficient mice fed a “Western-style diet” by increasing PPAR α and γ expression and reducing TC, MMP-9, and Cat S levels. Acta Pharmacol. Sin..

[69] Balakumar P., Mahadevan N. (2012). Interplay between statins and PPARs in improving cardiovascular outcomes: A double-edged sword?. Br. J. Pharmacol..

[70] Roy A., Jana M., Kundu M., Corbett G.T., Rangaswamy S.B., Mishra R.K., Luan C.H., Gonzalez F.J., Pahan K. (2015). HMG-CoA Reductase Inhibitors Bind to PPARα to Upregulate Neurotrophin Expression in the Brain and Improve Memory in Mice. Cell Metab..

[71] Singh K.D., Unal H., Desnoyer R., Karnik S.S. (2019). Mechanism of Hormone Peptide Activation of a GPCR: Angiotensin II Activated State of AT1R Initiated by van der Waals Attraction. J. Chem. Inf. Modeling.

[72] Wingler L.M., McMahon C., Staus D.P., Lefkowitz R.J., Kruse A.C. (2019). Distinctive Activation Mechanism for Angiotensin Receptor Revealed by a Synthetic Nanobody. Cell.

[73] Zou Y., Akazawa H., Qin Y., Sano M., Takano H., Minamino T., Makita N., Iwanaga K., Zhu W., Kudoh S. (2004). Mechanical stress activates angiotensin II type 1 receptor without the involvement of angiotensin II. Nat. Cell Biol..

[74] Barauna V.G., Magalhaes F.C., Campos L.C., Reis R.I., Kunapuli S.P., Costa-Neto C.M., Miyakawa A.A., Krieger J.E. (2013). Shear stress-induced Ang II AT1 receptor activation: G-protein dependent and independent mechanisms. Biochem. Biophys. Res. Commun..

[75] Mitra A.K., Gao L., Zucker I.H. (2010). Angiotensin II-induced upregulation of AT(1) receptor expression: Sequential activation of NF-kappaB and Elk-1 in neurons. Am. J. Physiol. Cell Physiol..

[76] Lin C.-P., Huang P.-H., Lai C.F., Chen J.-W., Lin S.-J., Chen J.-S. (2015). Simvastatin Attenuates Oxidative Stress, NF-κB Activation, and Artery Calcification in LDLR-/- Mice Fed with High Fat Diet via Down-regulation of Tumor Necrosis Factor-α and TNF Receptor 1. PLoS ONE.

[77] Hilgendorff A., Muth H., Parviz B., Staubitz A., Haberbosch W., Tillmanns H., Hölschermann H. (2003). Statins differ in their ability to block NF-kappaB activation in human blood monocytes. Int. J. Clin. Pharmacol. Ther..

[78] Banach-Orłowska M., Wyszyńska R., Pyrzyńska B., Maksymowicz M., Gołąb J., Miączyńska M. (2019). Cholesterol restricts lymphotoxin β receptor-triggered NF-κB signaling. Cell Commun. Signal..

[79] Zhang Z., Li Z., Cao K., Fang D., Wang F., Bi G., Yang J., He Y., Wu J., Wei Y. (2017). Adjunctive therapy with statins reduces residual albuminuria/proteinuria and provides further renoprotection by downregulating the angiotensin II-AT1 pathway in hypertensive nephropathy. J. Hypertens..

[80] Marino F., Guasti L., Cosentino M., Rasini E., Ferrari M., Maio R.C., Loraschi A., Cimpanelli M.G., Schembri L., Legnaro M. (2008). Simvastatin treatment in subjects at high cardiovascular risk modulates AT1R expression on circulating monocytes and T lymphocytes. J. Hypertens..

[81] Bai H.Y., Mogi M., Nakaoka H., Kan-No H., Tsukuda K., Wang X.L., Shan B.S., Kukida M., Yamauchi T., Higaki A. (2016). Synergistic Inhibitory Effect of Rosuvastatin and Angiotensin II Type 2 Receptor Agonist on Vascular Remodeling. J. Pharmacol. Exp. Ther..

[82] Suski M., Gębska A., Olszanecki R., Stachowicz A., Uracz D., Madej J., Korbut R. (2014). Influence of atorvastatin on angiotensin I metabolism in resting and TNF-α-activated rat vascular smooth muscle cells. J. Renin Angiotensin Aldosterone Syst..

[83] Tikoo K., Patel G., Kumar S., Karpe P.A., Sanghavi M., Malek V., Srinivasan K. (2015). Tissue specific up regulation of ACE2 in rabbit model of atherosclerosis by atorvastatin: Role of epigenetic histone modifications. Biochem. Pharmacol..

[84] Li Y.-H., Wang Q.-X., Zhou J.-W., Chu X.-M., Man Y.-L., Liu P., Ren B.-B., Sun T.-R., An Y. (2013). Effects of rosuvastatin on expression of angiotensin-converting enzyme 2 after vascular balloon injury in rats. J. Geriatr. Cardiol..

[85] Schindler C., Guenther K., Hermann C., Ferrario C.M., Schroeder C., Haufe S., Jordan J., Kirch W. (2014). Statin treatment in hypercholesterolemic men does not attenuate angiotensin II-induced venoconstriction. PLoS ONE.

[86] Patel V.B., Bodiga S., Fan D., Das S.K., Wang Z., Wang W., Basu R., Zhong J., Kassiri Z., Oudit G.Y. (2012). Cardioprotective effects mediated by angiotensin II type 1 receptor blockade and enhancing angiotensin 1-7 in experimental heart failure in angiotensin-converting enzyme 2-null mice. Hypertension.

[87] Hannich M., Nauck H.W.M., Reincke M., Adolf C., Völzke H., Rettig R., Hannemann A. (2018). Physiological Aldosterone Concentrations Are Associated with Alterations of Lipid Metabolism: Observations from the General Population. Int. J. Endocrinol..

[88] Baudrand R., Pojoga L.H., Vaidya A., Garza A.E., Vöhringer P.A., Jeunemaitre X., Hopkins P.N., Yao T.M., Williams J., Adler G.K. (2015). Statin Use and Adrenal Aldosterone Production in Hypertensive and Diabetic Subjects. Circulation.

[89] Hornik E.S., Altman-Merino A.E., Koefoed A.W., Meyer K.M., Stone I.B., Green J.A., Williams G.H., Adler G.K., Williams J.S. (2020). A clinical trial to evaluate the effect of statin use on lowering aldosterone levels. BMC Endocr. Dis..

[90] Drapala A., Sikora M., Ufnal M. (2014). Statins, the renin-angiotensin-aldosterone system and hypertension—A tale of another beneficial effect of statins. J. Renin Angiotensin Aldosterone Syst. JRAAS.

[91] Pignatelli P., Carnevale R., Pastori D., Cangemi R., Napoleone L., Bartimoccia S., Nocella C., Basili S., Violi F. (2012). Immediate antioxidant and antiplatelet effect of atorvastatin via inhibition of Nox2. Circulation.

[92] Santos M.T., Fuset M.P., Ruano M., Moscardó A., Valles J. (2009). Effect of atorvastatin on platelet thromboxane A(2) synthesis in aspirin-treated patients with acute myocardial infarction. Am. J. Cardiol..

[93] Undas A., Siudak Z., Topór-Mądry R., Leśniak M., Tracz W. (2012). Simvastatin administration reduces thromboxane production in subjects taking aspirin: Links between aspirin resistance and thrombin generation. Int. J. Cardiol..

[94] Millar Philip J., Floras John S. (2013). Statins and the autonomic nervous system. Clin. Sci..

[95] Lewandowski J., Symonides B., Gaciong Z., Siński M. (2015). The effect of statins on sympathetic activity: A meta-analysis. Clin. Auton. Res..

[96] Moreira E.D., Mostarda C.T., Moraes-Silva I.C., Ferreira J.B., Santos F.D., Lacchini S., De Angelis K., Rodrigues B., Irigoyen M.C. (2013). Effect of simvastatin in the autonomic system is dependent on the increased gain/sensitivity of the baroreceptors. Physiol. Rep..

[97] Chen Y., Michaelis M., Janig W., Devor M. (1996). Adrenoreceptor subtype mediating sympathetic-sensory coupling in injured sensory neurons. J. Neurophysiol..

[98] Pleiner J., Schaller G., Mittermayer F., Zorn S., Marsik C., Polterauer S., Kapiotis S., Wolzt M. (2004). Simvastatin prevents vascular hyporeactivity during inflammation. Circulation.

[99] Mühlhäuser U., Zolk O., Rau T., Münzel F., Wieland T., Eschenhagen T. (2006). Atorvastatin desensitizes β-adrenergic signaling in cardiac myocytes via reduced isoprenylation of G-protein γ-subunits. FASEB J..

[100] Tennakoon M., Kankanamge D., Senarath K., Fasih Z., Karunarathne A. (2019). Statins perturb Gβγ signaling and cell behaviors in a Gγ subtype dependent manner. Mol. Pharmacol..

[101] Ito M., Adachi T., Pimentel D.R., Ido Y., Colucci W.S. (2004). Statins Inhibit β-Adrenergic Receptor–Stimulated Apoptosis in Adult Rat Ventricular Myocytes via a Rac1-Dependent Mechanism. Circulation.

[102] Kandasamy K., Prawez S., Choudhury S., More A.S., Ahanger A.A., Singh T.U., Parida S., Mishra S.K. (2011). Atorvastatin prevents vascular hyporeactivity to norepinephrine in sepsis: Role of nitric oxide and α1-adrenoceptor mRNA expression. Shock.

[103] Carillion A., Feldman S., Na N., Biais M., Carpentier W., Birenbaum A., Cagnard N., Loyer X., Bonnefont-Rousselot D., Hatem S. (2017). Atorvastatin reduces β-Adrenergic dysfunction in rats with diabetic cardiomyopathy. PLoS ONE.

[104] Vandresen-Filho S., França L.M., Alcantara-Junior J., Nogueira L.C., de Brito T.M., Lopes L., Junior F.M., Vanzeler M.L., Bertoldo D.B., Dias P.G. (2015). Statins enhance cognitive performance in object location test in albino Swiss mice: Involvement of beta-adrenoceptors. Physiol. Behav..

[105] Tokuhisa H., Murai H., Okabe Y., Hamaoka T., Sugimoto H., Mukai Y., Inoue O., Takashima S.-I., Kato T., Usui S. (2018). Differential effects of lipophilic and hydrophilic statins on muscle sympathetic nerve activity in heart failure with preserved left ventricular ejection fraction. Auton. Neurosc..

[106] Gorabi A.M., Kiaie N., Bianconi V., Jamialahmadi T., Al-Rasadi K., Johnston T.P., Pirro M., Sahebkar A. (2020). Antiviral effects of statins. Progress Lipid Res..

[107] Lee H.-Y., Ahn J., Park J., Kyung Kang C., Won S.-H., Kim D.W., Park J.-H., Chung K.-H., Joh J.-S., Bang J.H. (2021). Beneficial Effect of Statins in COVID-19–Related Outcomes—Brief Report: A National Population-Based Cohort Study. Arterioscler. Thromb. Vasc. Biol..

[108] Gupta A., Madhavan M.V., Poterucha T.J., DeFilippis E.M., Hennessey J.A., Redfors B., Eckhardt C., Bikdeli B., Platt J., Nalbandian A. (2021). Association between antecedent statin use and decreased mortality in hospitalized patients with COVID-19. Nat. Commun..

[109] Zhang X.-J., Qin J.-J., Cheng X., Shen L., Zhao Y.-C., Yuan Y., Lei F., Chen M.-M., Yang H., Bai L. (2020). In-hospital use of statins is associated with a reduced risk of mortality among individuals with COVID-19. Cell Metab..

[110] Namsolleck P., Moll G.N. (2020). Does activation of the protective Renin-Angiotensin System have therapeutic potential in COVID-19?. Mol. Med..

[111] Meng J., Xiao G., Zhang J., He X., Ou M., Bi J., Yang R., Di W., Wang Z., Li Z. (2020). Renin-angiotensin system inhibitors improve the clinical outcomes of COVID-19 patients with hypertension. Emerg. Microbes Infect..

[112] D'Ardes D., Boccatonda A., Rossi I., Guagnano M.T., Santilli F., Cipollone F., Bucci M. (2020). COVID-19 and RAS: Unravelling an Unclear Relationship. Int. J. Mol. Sci..

[113] Hoffmann M., Kleine-Weber H., Schroeder S., Krüger N., Herrler T., Erichsen S., Schiergens T.S., Herrler G., Wu N.-H., Nitsche A. (2020). SARS-CoV-2 cell entry depends on ACE2 and TMPRSS2 and is blocked by a clinically proven protease inhibitor. Cell.

[114] Verdecchia P., Cavallini C., Spanevello A., Angeli F. (2020). COVID-19: ACE2centric infective disease?. Hypertension.

[115] Verdecchia P., Cavallini C., Spanevello A., Angeli F. (2020). The pivotal link between ACE2 deficiency and SARS-CoV-2 infection. Eur. J. Intern. Med..

[116] Vicenzi M., Di Cosola R., Ruscica M., Ratti A., Rota I., Rota F., Bollati V., Aliberti S., Blasi F. (2020). The liaison between respiratory failure and high blood pressure: Evidence from COVID-19 patients. Eur. Respir. J..

[117] Burrell L.M., Johnston C.I., Tikellis C., Cooper M.E. (2004). ACE2, a new regulator of the renin–angiotensin system. Trends Endocrinol. Metab..

[118] Michaud V., Deodhar M., Arwood M., Al Rihani S.B., Dow P., Turgeon J. (2020). ACE2 as a Therapeutic Target for COVID-19; Its Role in Infectious Processes and Regulation by Modulators of the RAAS System. J. Clin. Med..

[119] Subir R., Jagat J.M., Kalyan K.G. (2020). Pros and cons for use of statins in people with coronavirus disease-19 (COVID-19). Diabetes Metab. Syndr..

[120] Tan W.Y.T., Young B.E., Lye D.C., Chew D.E.K., Dalan R. (2020). Statin use is associated with lower disease severity in COVID-19 infection. Sci. Rep..

[121] Sun T., Jiang D., Zhang L., Su Q., Mao W., Jiang C. (2016). Expression profile of cathepsins indicates the potential of cathepsins B and D as prognostic factors in breast cancer patients. Oncol. Lett..

[122] Altaf A., Qu P., Zhao Y., Wang H., Lou D., Niu N. (2015). NLRP3 inflammasome in peripheral blood monocytes of acute coronary syndrome patients and its relationship with statins. Coron. Artery Dis..

[123] Hurks R., Hoefer I.E., Vink A., Pasterkamp G., Schoneveld A., Kerver M., de Vries J.P.P.M., Tangelder M.J., Moll F.L. (2010). Different Effects of Commonly Prescribed Statins on Abdominal Aortic Aneurysm Wall Biology. Eur. J. Vasc. Endovasc. Surg..

[124] Abisi S., Burnand K.G., Humphries J., Waltham M., Taylor P., Smith A. (2008). Effect of statins on proteolytic activity in the wall of abdominal aortic aneurysms. Br. J. Surg..

[125] Smith R., Solberg R., Jacobsen L.L., Voreland A.L., Rustan A.C., Thoresen G.H., Johansen H.T. (2014). Simvastatin Inhibits Glucose Metabolism and Legumain Activity in Human Myotubes. PLoS ONE.

[126] Zhang J., Shi X., Hao N., Chen Z., Wei L., Tan L., Chen Y., Feng H., Chen Q., Zhu G. (2018). Simvastatin Reduces Neutrophils Infiltration Into Brain Parenchyma After Intracerebral Hemorrhage via Regulating Peripheral Neutrophils Apoptosis. Front. Neurosci..

[127] Chello M., Anselmi A., Spadaccio C., Patti G., Goffredo C., Di Sciascio G., Covino E. (2007). Simvastatin Increases Neutrophil Apoptosis and Reduces Inflammatory Reaction After Coronary Surgery. Ann. Thoracic Surg..

[128] Yamamoto N., Fujii Y., Kasahara R., Tanida M., Ohora K., Ono Y., Suzuki K., Sobue K. (2016). Simvastatin and atorvastatin facilitates amyloid β-protein degradation in extracellular spaces by increasing neprilysin secretion from astrocytes through activation of MAPK/Erk1/2 pathways. Glia.

